# The effects of an adapted mental health literacy curriculum for secondary school students in Germany on mental health knowledge and help-seeking efficacy: results of a quasi-experimental pre-post evaluation study

**DOI:** 10.3389/fpubh.2023.1219925

**Published:** 2023-08-16

**Authors:** Alexandra Maria Freţian, Sandra Kirchhoff, Ullrich Bauer, Orkan Okan

**Affiliations:** ^1^Faculty of Educational Science, Bielefeld University, Bielefeld, Germany; ^2^Department of Sport and Health Sciences, Technical University of Munich, Munich, Germany

**Keywords:** mental health literacy, school intervention, adolescents, mental health knowledge, help-seeking efficacy

## Abstract

**Background:**

Because the majority of mental illnesses develop early in life, effective preventative public mental health interventions are needed. Interventions fostering mental health literacy can be used to enhance personal resources and capacities to facilitate mental health care and thus, address help-seeking barriers. A Canadian mental health literacy school curriculum was adapted, piloted, and evaluated for the use in German schools. The study presents the intervention’s effects on mental health knowledge and help-seeking efficacy among 10^th^ grade students in Germany.

**Methods:**

10^th^ grade students (aged 14–17 years old) from one secondary school participated in a pre- and post-intervention control group study. Both groups completed a questionnaire at two time points assessing mental health knowledge and help-seeking efficacy. Repeated measure analysis of variance (ANOVA) was employed to evaluate the intervention’s effects.

**Results:**

Data from 188 students was eligible for analysis. The analysis of the baseline data reveals a high comparability of the two groups in terms of demographics, and initial mental health knowledge and help-seeking efficacy scores. ANOVA results showed significant improvements for the intervention group having a large effect size for mental health knowledge (*f* = 0.574, *p* < 0.001, partial η^2^ = 0.25) and a medium effect size for help-seeking efficacy (*f* = 0.311, *p* < 0.001, partial η^2^ = 0.09).

**Conclusion:**

The first-time application and evaluation of an adapted mental health literacy school curriculum shows significant increases in mental health knowledge and help-seeking efficacy, two core dimensions of mental health literacy, among 10^th^ grade students in Germany. Further studies are needed to confirm these results as well as have a more in-depth analysis on the interrelations of the different dimensions of mental health knowledge and help-seeking practices.

## 1. Introduction

Worldwide, mental illness is among the leading causes of disability, and particularly so for those under the age of 25. In high income countries mental illness is the leading cause of both, years of life prematurely lost (mainly due to suicide) and years lived with disability (1). Adolescence is a very sensitive time period for addressing mental illness, as it is the time span when most disorders emerge, about 50% of them developing before the age of 14 (2).

It has been observed that when a mental health problem arises, help-seeking rates are generally low, especially among children and adolescents (3). Counterintuitively, the earlier mental health problems arise, the more likely it is that they remain untreated for longer periods of time (4). This is not only detrimental in terms of the distress and impairment associated with current mental health status, but also in terms of the future success of therapy. The absence of treatment has been associated with worsening of mental illness symptomatology^1^ and a higher likelihood of a chronic dysfunction (5). Thus, improving the timely use of mental health care is crucial for impeding the progression of an existing condition and the onset of a new one (6).

On the one hand factors hindering structural change need to be addressed when advocating for the improvement of mental health care access. It has been stated that mental health system responses, especially among children and adolescents have been inadequate, and need to be updated (6). Appropriate mental health care needs to be age-appropriate and provided by multidisciplinary teams (6). Moreover, costs and availability of mental health care services need to be considered in order to make it more accessible for young people with the highest needs. Those experiencing poverty, abuse, and violence early in life have an increased risk of developing mental health problems (7). It has been stressed that the increased risk of developing a mental illness for children living in poverty works through both, direct and indirect mechanisms, such as material hardship^2^ and their negative impacts on cognitive and social–emotional development, as well as parental stress resulting in limited resources to provide proper caregiving, respectively (8). Thus, another preventative access point is to tackle the social determinants of poor mental health, and then offer material and psycho-social support for families (9).

On the other hand, underlying factors of individual help-seeking behavior should be concomitantly considered in order to improve access to mental health care. A systematic literature review on barriers of young peoples’ help-seeking of formal mental health care identified stigma, knowledge, and the perceptions of help-seeking and therapy to be the most relevant impediments (followed by structural issues such as availability and costs related to mental health care) (10). The majority of programs that focus on altering individual modifiable factors that facilitate help-seeking, intervene at the level of parental behaviors or attitudes, leaving young people, themselves, out of the equation (11). While engaging parents is a relevant strategy, especially among younger age groups, a targeted approach would be beneficial for young people, themselves, as long as it addresses hindering and facilitating factors related to help-seeking such as the stigma surrounding mental illness (12), knowledge of mental health problems, and young people’s preference for self-reliance (3, 13).

When considering individual aspects that promote mental health help-seeking, the concept of mental health literacy (MHL) is helpful as it refers to a set of knowledge, attitudes, and skills related to mental health (14). MHL has been defined to include the following components: “understanding how to obtain and maintain positive mental health; understanding mental disorders and their treatments; decreasing stigma related to mental disorders; and, enhancing help-seeking efficacy (knowing when and where to seek help and developing competencies designed to improve one’s mental health care and self-management capabilities)” (14). Given its multidimensional structure, the relationship between each facet and help-seeking can be discussed separately. For example, there is empirical evidence of a positive association between the ability to recognize a mental illness (based on the description of symptomatology) and positive attitudes towards formal mental health help-seeking (15–17) as well as to actual use of therapeutic care (18). However, the literature holds conflicting findings in this regard as one study showed that the ability to identify a mental health problem has a negative correlation with help-seeking intentions, while knowledge of treatment efficacy is positively linked to help-seeking intentions (19). Thus, different knowledge domains can either have a negative or positive association to help-seeking intentions. The authors discuss the potential role that stigma might play, when it comes to labeling mental disorders. Because labels of mental illness are known to have stigma attached to them, the recognition and labeling process might lead to reluctance to seek help if stigma is not addressed concomitantly (19).

While some associations between particular dimensions of MHL are well established, a comprehensive interaction model on how all aspects influence each other as well as their direct or indirect link to help-seeking behavior is lacking (19, 20). With the concept of MHL in focus, a series of reviews have summarized the effectiveness of interventions for young people that aim to improve mental health knowledge and attitudes related to mental health and help-seeking intentions or behaviors. A meta-analysis of MHL interventions for adolescents concluded that the greatest change was found within the knowledge dimension (21). The change reached a moderate effect size, which remained stable at an average of 5 months follow-up assessment (21). Similarly, another review concluded that the most stable results could be observed for mental health knowledge, while for other dimensions of MHL the results are less conclusive, with a negligible magnitude in change (22). When focusing on help-seeking as an outcome of school-based interventions, a review has found small improvements for individual help-seeking as well as help-seeking for others, that remain observable after a three to 6 month follow-up period (23). These and other reviews on MHL interventions generally conclude that there is a variability in the effectiveness of individual studies, depending on various factors such as study design and quality (e.g., risk of bias, methodologically sound measurements), and characteristics of the intervention (e.g., type, length, setting) (21, 22, 24, 25).

In order to strengthen the evidence based on the effectiveness of an already-existing intervention program, entitled “The Mental Health and High School Curriculum Guide (MHC),” an evaluation study was conducted in Germany. This publication reports the findings of the first-time application and evaluation of the MHC, which was translated and adapted to be applied in German schools. The scope of the evaluation study is to verify whether this MHL resource is suitable for improving the mental health knowledge and help-seeking efficacy of 10^th^ grade students in Germany. Other publications have an in-depth focus on the adapted MHC’s effects on stigmatizing attitudes (26) and its acceptance by the participating students (27).

## 2. Methods

### 2.1. Sample

Overall, the whole sample consisted of data from 297 students attending four schools, including three secondary schools and one vocational school. The different school forms led to great variability in terms of age (from 14 to 21), language proficiency, and implementation method (during a project day or within weekly school classes). The schools that implemented the MHC in a weekly fashion, faced interruptions due to COVID-19 related school closings, that lead to incomplete implementation of the modules and/or an extension of the post-evaluation time point.^3^ Since all these factors would lead to a huge variability and bias in the implementation and evaluation process, we chose to use a partial sample from one school only where the MHC was implemented throughout the whole 10^th^ grade as a project day. The partial sample consisted of 216 10^th^ graders recruited from one school in Bielefeld, Germany. At pretest 204 participants filled out the questionnaire, while at posttest 200 participants completed the questionnaire. The same 188 students filled out the questionnaire at both time points. All upcoming information about the intervention procedure and data analysis are based on the partial sample.

### 2.2. Procedure

Our research team conducted the German translation of the MHC, as part of the “IMPRES” project [a subproject of the “Health Literacy in Childhood and Adolescence” research consortium (28)]. A local cooperation group was established together with the regional school board and mental health care coordination, an NGO with lived experience of mental illness, and a non-profit foundation, in order to help promote the implementation in local schools.

The MHC intervention was implemented as a pre- and post-test control group design. In fall 2020 five 10^th^ grade classes of one school received the German version of the MHC within 1 day. Data was collected about 1 week before (pre-test) and 1 week after the intervention (post-test) via paper-pencil questionnaires. The next term of 10^th^ graders functioned as a control group in which a similar data collection period between one and two weeks was applied. In fall 2021, they completed the paper-pencil questionnaires on two different occasions, about one to 2 weeks apart. The control group received the intervention at a later time point. In both cases the intervention was carried out within the same day.

Four religion teachers and a school counselor^4^ were in charge of teaching the modules during the project day. A one-day training was provided for school personnel interested in implementing the MHC. Because not all teachers participated in the training, the ones present were instructed to adhere to the described lesson plans and to brief their colleagues about the use of the materials. While on the one hand, the teachers were instructed to implement the core materials in a standardized way (as described in the lesson plans), they were, on the other hand given pedagogical freedom to adapt content to their students’ needs.

Additionally, the teachers who implemented the intervention in their classrooms were asked to fill out a brief questionnaire about the circumstances of implementation. All teachers reported that no other mental health interventions have been carried out in their schools. Nobody used any additional materials, and, for the most part, all recommended lessons were implemented.

In order to implement the module regarding lived experience about mental illness a presentation of personal experience was provided either by a presenter in the classroom or via a pre-recorded video. The presenters who visited the schools were experts with lived experience of mental illness and most were affiliated with the collaborating local NGO. A training as well as opportunities for peer-support and exchange were provided to potential presenters before and throughout their participation in the intervention. In the training, the presenters were familiarized with the core contents of the MHC and received recommendations on the outline of their presentation (e.g., description of the symptoms of illness and the impact on every-day life, the treatment process, the management of the illness and helpful experiences in the healing process). The participating schools could choose between showing videos or having a presenter in their classroom. The school participating in the evaluation preferred to have presenters visit their school, however, because the intervention took place on the same day with five different classes, only one presenter was available at that time and, thus, was only able to visit two of the five classes.

### 2.3. The intervention program

The school-based MHL intervention, entitled “The Mental Health and High School Curriculum Guide” was previously evaluated and used in cross national contexts. So far, the MHC has proven to significantly improve mental health knowledge and stigmatizing attitudes directly after its application (29, 30), at a 2 months – (31, 32) as well as a 1 year follow-up (33). Fewer studies have focused on its effectiveness in terms of improving help-seeking efficacy. While one report and one randomized controlled study show benefits (30, 34) another study found no improvements in help-seeking intentions (35). The MHC is an evidence-based teaching manual for improving MHL of students and teachers alike (36). Developed in Canada, this program was created as a resource to be used in the classroom by school staff. It primarily targets grades nine and ten (ages 13 to 15). The MHC comprises a total of six modules on the following topics: 1) destigmatizing mental illnesses 2) understanding mental health and mental illness, 3) information regarding specific mental illnesses (e.g., symptoms, treatment, risk factors) 4) lived experiences of mental illness 5) receiving help and support, and 6) positive mental health. The MHC contains activities and materials that can be implemented based on an outlined step-by-step lesson plan in about six to 12  hours of teaching. Additionally, a teacher’s study guide helps navigate the materials, and provides additional background information on the topics of mental health. The activities contain both, individual and group-work elements and rely on digital (presentation slides, videos, etc.) as well as printed materials (activity cards, information sheets, etc.).

The main adaptations were related to reducing the overall implementation time. Parts of some activities were left out or marked as “optional” in the German version of the MHC, thus requiring an overall implementation time of at least seven to eight school hours. The changes and cutbacks in the activities mostly regarded the functioning and role of the brain. This adaptation was briefed with and approved by the Canadian creators. In order to maintain the quality of the intervention, the core content was not changed.

### 2.4. Ethical approval and data protection

Ethical clearance was obtained for data collection by the ethics committee of Bielefeld University (reference number EUB 2019–145). No incentives were used for participation. Participation was voluntary and could be withdrawn anytime without providing reasons. Written informed consent was collected from students and, additionally, from parents or legal guardians of students younger than 16 years of age. The school principal and the respective teachers implementing the intervention agreed to hand out the informed consent forms for participating in the study to the parents or guardians and students.

Each participating student received a personal numerical code in order to be able to connect the responses from the first and second measurement time point. Only the appointed data custodian was able to connect the codes to the names of the participants. This was only possible up until the second measurement time point, after which the according information connecting the names and codes of the participants was destroyed.

### 2.5. Measures

Mental health knowledge and help-seeking efficacy were the outcomes of interest. The scales assessing these outcomes were available in English and translated into German by a bilingual research team. To ensure the quality of the translation, a research assistant independently translated the items back to English, and further language adjustments were made. The reliability (Cronbach’s Alpha) of each scale was computed at baseline and post-test.

#### 2.5.1. Mental health knowledge (MHK)

A 30-item scale was used to measure knowledge along the following domains corresponding to the content of the six modules of the MHC: an understanding of the functioning of the brain, symptoms of common mental disorders, coping with stress and positive mental health, causes of mental disorders and treatment, and suicide. The response format of the items were presented as “true,” “false,” or “do not know.” One item about the brain functioning was replaced with an item about potential effects of stress on the brain from the “Knowledge and Attitudes to Mental Health (KAMH)” scale (37, 38). A second item regarding medication usefulness was slightly adapted to make it more generally applicable (see Supplementary Table S1). This change was motivated by the underlying adaptation of the MHC where less emphasis was put on detailed brain functioning. A sum score was calculated by assigning one point to each correct answer, while the “do not know” response was coded with zero points along with the incorrect answers. A higher sum score represents better mental health knowledge. Only if participants completed all 30 items was the sum score calculated. Possible sum scores range from zero to 30. In previous studies the internal consistency of mental health knowledge items was α = 0.85–0.88 (33).

#### 2.5.2. Help-seeking efficacy (HSE)

A five-item measure was used to estimate students’ efficacy of seeking and recommending psychological help when suspecting a psychological problem. The answer options were on a seven-point Likert scale ranging from “strongly disagree” to “strongly agree.” A sum score was calculated if participants provided answers to all items. Higher scores reflect a higher help-seeking efficacy, with a possible score range between five and 35. The scale has been validated with a sample of teachers, where it has reached acceptable internal consistency (α = 0.78) (39). The original items together with the German translation are shown in Supplementary Table S2.

### 2.6. Analysis

Only participants that completed the questionnaire on both the first and second measurement time points were included in the analysis (*N* = 188). Due to the nested structure of the data, we verified whether the outcomes of interest showed a clustering effect in regard to class affiliation by calculating the intraclass correlation coefficient (ICC). Because the ICC turned out to be non-significant for both dependent variables, the need for multilevel modeling was ruled out, and we, therefore, used traditional analysis techniques.

Descriptive analysis was used to provide an overview of the characteristics of the study participants. Moreover, chi-square-tests and two-sample t-tests were used to verify whether the control and intervention group differed in terms of age, gender, parental migration background, contact to someone with a mental illness, and MHK as well as and HSE baseline scores.

The change in MHK was reported through the percentage of correctly answered items at baseline and at post-test both at item-level and for the overall sum score. For each item, a paired sample t-test was performed to verify whether the change was statistically significant. The same procedure was used for the items of the HSE scale. In order to verify the impact of the intervention on MHK and HSE, a repeated measures ANOVA was used and corresponding effect sizes were calculated. The significance level was set at α = 0.05. The Statistical Package for the Social Sciences (SPSS) version 22 was used to analyze the data.

## 3. Results

The intervention group (IG) consisted of 122 participants, out of which 86.9% completed the questionnaire at both times, and the control group (CG) consisted of 94 participants, out of which 87.2% had data from the two time points. In the IG, three classes received the video as part of one module, and in two classes, a speaker with lived experience of mental illness held a presentation.

### 3.1. Sample description – baseline scores

Table 1 shows the distribution of demographic characteristics as well as the MHK and HSE scores at baseline for the whole sample, and separately for the IG and CG. Only one significant difference between IG and CG was found in terms of age where the CG was slightly older. No significant differences were found at baseline for MHK and HSE.

**Table 1:** Distribution of sample characteristics for total sample, IG, and CG.

	Total	Intervention	Control	Test statistics (*t*-test, Chi Square), *p*-values
Gender *n* (%)	98.4%, 185	99.1%, 105	97.6%, 80	*X*^2^ (1, *N* = 184) = 0.099, p = .753^a^
Female	58.4%, 108	57.1%, 60	60%, 48	
Male	41.1%, 76	41.5%, 44	40%, 32	
Other	0.5%, 1	1%, 1	–	
Parental migration background %, *n*	99.5%, 187	100%, 106	98.8%, 81	*X*^2^ (2, *N* = 187) = 0.881, *p* = 0.644
Both parents	25.1%, 47	25.5%, 27	24.7%, 20	
One parent	12.3%, 23	14.2%, 15	9.9%, 8	
No migration background	62.6%, 117	60.4%, 64	65.4%, 53	
Contact with mental illness %, *n*	100%, 188	100%, 106	100%, 82	*X*^2^ (1, *N* = 188) = 0.644, *p* = .412^b^
Yes	60.6%, 114	63.2%, 67	57.3%, 47	
Do not know	17%, 32	14.2%, 15	20.7%, 17	
Do not want to answer	3.2%, 6	4.7%, 5	1.2%, 1	
No	19.1%, 36	17.9%, 19	20.7%, 17	
Age, M (SD) *n*	15.14 (0.56), 187	15.01 (0.56) 106	15.31 (0.52) 81	t (185) = −3.743, *p* < 0.001
Sum score MHK T1^c^, M (SD), *n*	17.07 (4.04), 174	17 (3.75) 99	17.2 (4.42) 75	t (172) = −0.258, *p* = 0.797
Sum score HSE T1^c^, M (SD), *n*	26.36 (4.26), 186	26.47 (3.99) 106	26.21 (4.61) 80	t (184) = 0.410, *p* = 0.682

a Comparison between male and female.

b Comparison between “yes” and the other categories combined.

c First measurement time point.

### 3.2. Changes in MHK and HSE

**Figure 1:**
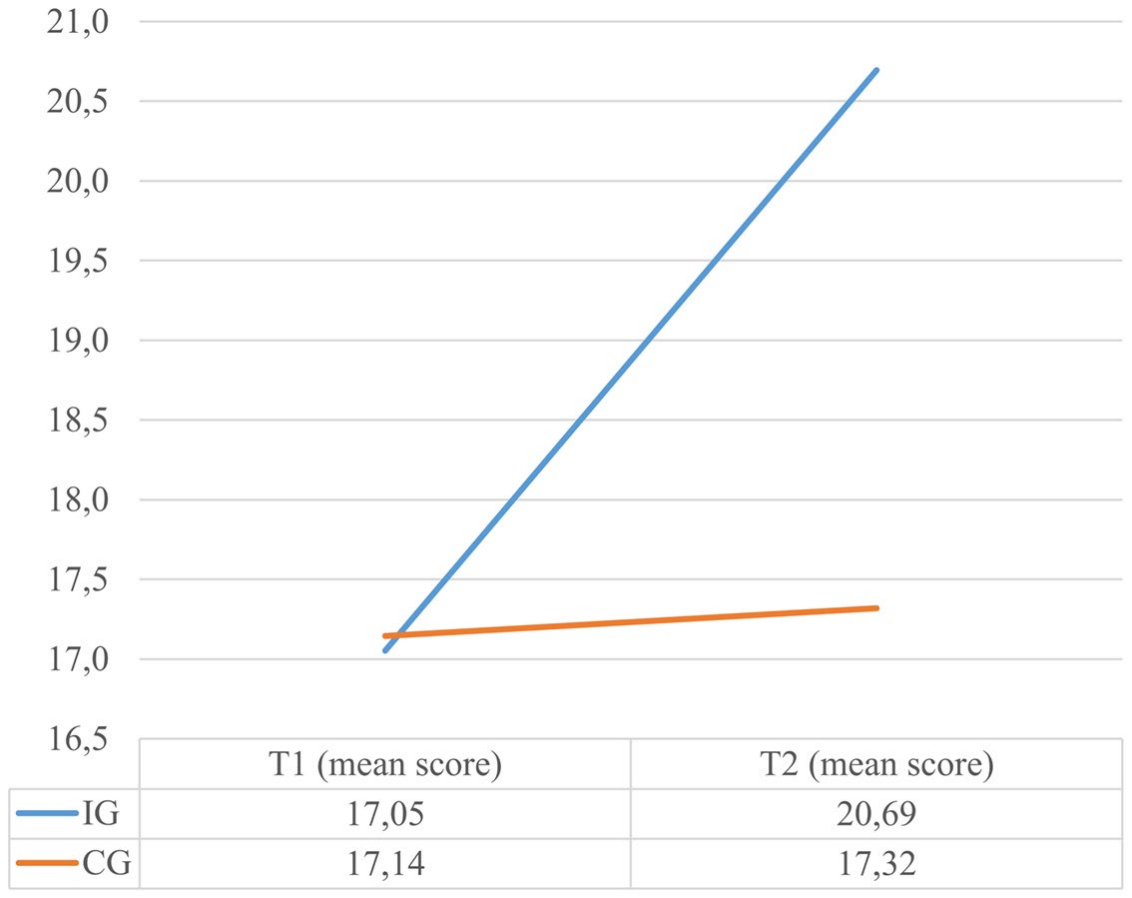
The change in MHK from T1 to T2 for the IG and CG.

**Figure 2:**
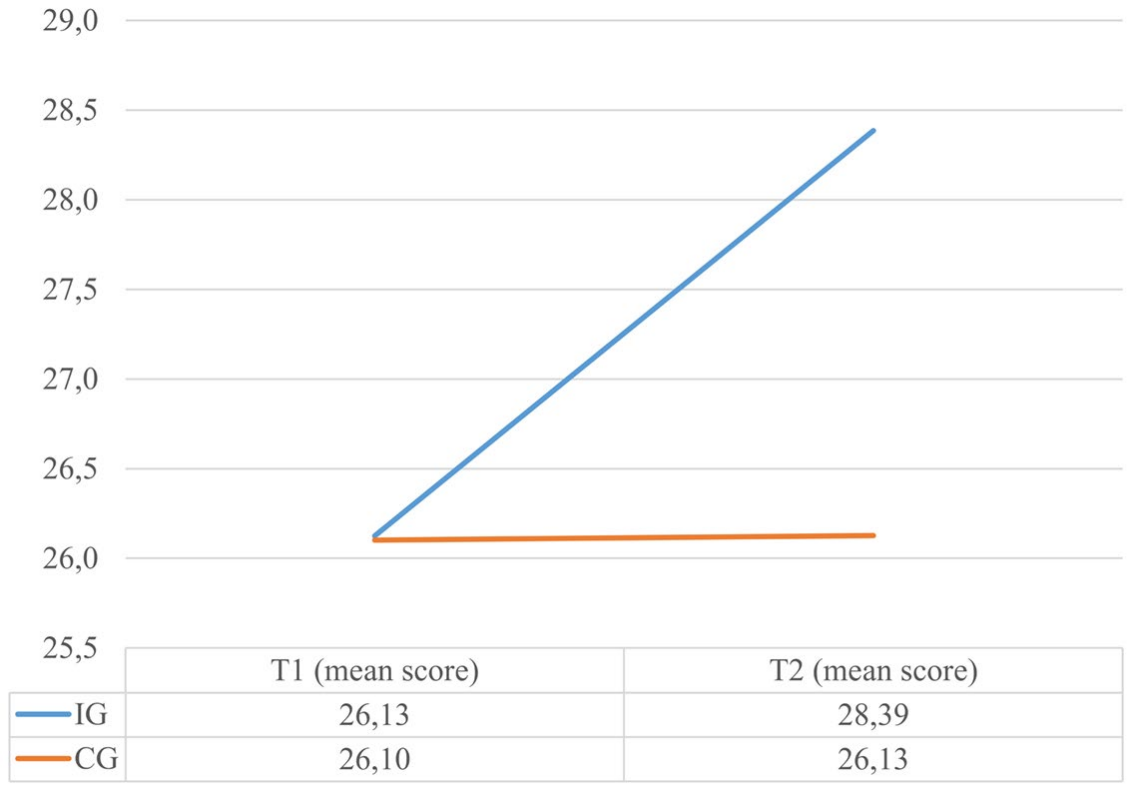
The change in HSE from T1 to T2 for the IG and CG.

The results of the conducted repeated measures ANOVA evaluating the effects of the adapted MHC on MHK and HSE can be retrieved from Table 2. While at pretest, the IG answered 56.9% of the items on the MHK questionnaire correctly, at post-test the percentage increased to 69%. This constitutes a significant change with a large effect size. In contrast, no significant change was noted for the CG. The change in the MHK sum scores can be seen in Figure 1. The change in HSE was significant with a medium effect size and can be viewed in Figure 2. In the IG the mean score of the HSE scale increased from 26.1 to 28.4 points, while in the CG it remained unchanged at 26.1 points. The MHK had an internal consistency of 0.69 at baseline and 0.79 at post-test, while the HSE had 0.62 at baseline and 0.69 at post-test. Supplementary Table S3 and Table 3 show the results of the changes for each item of the MHK and HSE questionnaire, separately for the IG.

**Table 2:** Repeated measure ANOVA for MHK and HSE.

Outcomes		*n*	Mean (SD)	Repeated measure ANOVA			
				*F*	*p*-values	Partial *η*^2^	Cohen’s *F*
Mental health knowledge (30 items)							
Intervention	Pre-test	95	17 (3.7)	53.43	< 0.001	0.248	0.574
	Post-test	95	20.7 (4.3)				
Control	Pre-test	69	17.1 (4.3)				
	Post-test	69	17.3 (4.5)				
Help-seeking efficacy (5 items)							
Intervention	Pre-test	96	26.1 (3.9)	16.76	< 0.001	0.088	0.311
	Post-test	96	28.4 (3.7)				
Control	Pre-test	79	26.1 (4.5)				
	Post-test	79	26.1 (4.5)				

**Table 3:** Results of paired sample *t*-tests for individual items of the HSE scale in the IG.

Help-seeking efficacy items	*N*	T1		T2		Paired-sample t-test
		M	SD	M	SD	
1. In general, asking for help for a mental health problem or disorder is helpful	106	6.10	1.29	6.62	0.83	t (105) = −4.243, *p* < 0.001
2. I am comfortable asking for help for a mental health problem or disorder	106	3.59	1.67	4.11	1.61	t (105) = −3.223, *p* = 0.002
3. If I think I may have a mental health problem or mental disorder (such as depression, social anxiety disorder, etc.), I will ask for help	106	4.00	1.70	4.77	1.66	t (105) = −4.755, *p* < 0.001
4. If I thought one of my friends or peers needed help with a mental health problem or disorder (such as depression), I would encourage them to seek help	106	6.33	0.84	6.45	0.82	t (105) = −1.184, *p* = 0.239
5. If I thought one of my family members needed help with a mental health problem or disorder (such as depression), I would encourage them to seek help	96	6.41	0.92	6.54	0.82	t (95) = −1.298, *p* = 0.197

In the IG, on the MHK scale, the biggest improvements can be observed in the categories symptoms of common mental disorders, coping with stress and positive mental health, and causes and treatments for mental illness. The highest magnitude in improvement could be seen in item number 2, “People who have mental illness can at the same time have mental health,” with an additional 38.1% correct answers after the intervention followed by item number 18, “Medicines should never be used to treat a mental disorder” with an improvement of 31.2%. Overall a significant improvement of knowledge could be observed in 17 of the 30 items. In the IG there were no items that significantly worsened after the intervention. Conversely, in the CG, significant changes were registered for three items, two of which worsened while one improved.

It can be noted that in both, the IG and CG, within the HSE scale, the area related to recommending help for friends or family as well as the areas related to positive attitudes towards help-seeking in general reach ceiling effects with a mean of at least 6.1 out of 7 points at baseline. Significant changes could be seen for the IG in three of the five items that focused on own help-seeking efficacy, but also a positive attitude about help-seeking in general, while no significant changes were found in the CG. The biggest significant improvement could be found in asking for help when thinking that one might have a mental health problem.

## 4. Discussion

This is the first German translation, adaptation, implementation, and evaluation of the MHC. The findings show large and significant changes in terms of knowledge as well as medium changes in terms of help-seeking efficacy among 10^th^ grade students in Germany.

The results in improving the knowledge domain are in line with previous findings such as one randomized controlled study conducted in Canada among 9th grade school students (31), and a controlled pre-post-follow-up evaluation carried out in schools and universities in Nicaragua (35) which yielded medium to substantively higher scores after the intervention. The baseline scores in our sample were higher than the ones of the students in Canada or Nicaragua, however, the program was still able to achieve improvements. While the evidence based on the MHC’s usefulness in improving knowledge and stigma, up to one year after the intervention, is well-established (33), less congruent findings have been reported for HSE (34, 35). While one study using the same measurement instrument as we did found improvements in HSE (34), another study which investigated help-seeking intentions and behaviors could not find significant changes (35). Furthermore, the German version of the MHC has also proven to significantly improve stigmatizing attitudes (26) and had a high acceptability among students (27). After being asked how they evaluated the program, they were encouraged to provide open-ended answers about their reasonings. The main topics that came up were in line with the key messages presented within the MHC: the importance of being informed about mental health, generally, as well as destigmatizing mental illness, and recognizing and knowing how to handle mental health problems, specifically (27).

While MHK and HSE can be viewed as entire constructs, one can apply a more in-depth analytical approach when considering the trends that become apparent at item-level. MHK can broadly be understood as knowledge related to distinct categories such as symptoms of common mental health disorders, coping with stress and positive mental health, etiology and risk factors of mental illness, and treatments for mental illness. MHK, and especially particular MHK categories such as recognizing mental illnesses and their symptoms as well as trusting therapeutic processes, have been identified as facilitating factors for help-seeking (10, 13). Thus, it is worthwhile to separately consider items of the MHK scale that are related to these particular domains. For example, it can be noted that some of the highest percentage changes brought on by the implementation of the MHC were related to knowledge about treatment. While initially less than two thirds (59%) stated that medication is helpful in the treatment of mental illness, after the intervention, the number increased to 91%. Moreover, an increase of 20.3 percentage points could be registered for the item regarding general treatment efficacy for mental illness (item number 11: “Most people who have a mental illness do not get well and stay well with treatment.”).

Further, in terms of knowledge about mental illness symptomatology, a more nuanced picture can be observed. While for common or well-known mental illnesses such as depression and anxiety, the correct labeling of associated symptoms was already rather high before the intervention (87.6 and 80.2%, respectively, answered correctly), whereas for eating disorders it was rather low, with only 23.6% providing the correct answer. Comparing these rates with those found in other studies, similarities as well as differences can be noted. For example, a vignette-based study among 13 to 16  year-olds found that the ability to recognize depression was highest with 82.2%, followed by psychosis with 68.7 and 62.3% for social phobia (40). Another study using vignette-based measures found significantly higher recognition for depression than for social anxiety, however, still only under 50% of participants identified the respective disorders correctly (41). Generally, a rather high variability can be found in terms of recognizing mental illnesses across populations. Thus the presentation of symptoms of various specific mental illnesses seems to be a useful part of a MHL program. The advantage of the MHC in this regard is that it provides elaborated materials for eight different mental illnesses and, thus, teachers can set emphasis on the illnesses that are rather unknown or of greater relevance to their students.

HSE encompasses attitudes towards help-seeking in general, own help-seeking and providing help for others. It is noteworthy that at baseline, the willingness to recommend help for others was already high, reaching ceiling effects. Even though the scores also increased at post-measurement, they did not reach a significant level. Other than the already high initial score, an explanation for the lack of significant change can be that this program is not specifically designed to improve help-seeking for others or that the items themselves cannot reliably depict this area. Future studies could verify this by using help-seeking scales with a broader scope. However, the attitudes towards seeking help for oneself and to seeking help in general were positively influenced by the MHC.

An open question for future studies is how the improved attitudes are associated to actual help-seeking behavior. In order to comprehensively answer the question of interrelation between intentions and behaviors, different theoretical models such as the theory of planned behavior (42), or theory of change (43) could be used. However, addressing these relations empirically requires that all mentioned constructs in those particular theories are measured. So far in MHL research, criticism regarding missing relevant outcome measures has been expressed for various constructs such as knowledge acquisition (44), but also for observable behavior such as use of therapy when displaying clinically relevant symptomatology. Moreover, the criticism has not only been addressed towards missing measurements, but also towards its lack of nuance in terms of differentiating between topics or dimensions (19). Thus, open questions remain as to what types of knowledge (e.g., regarding treatment, symptoms, or etiology of mental illness) are of particular relevance to promote help-seeking, and what other MHL dimensions drive or hinder this relationship. Thus, future studies, should also address other relevant mental health related outcomes (e.g., help-seeking behavior, well-being, etc.) and establish their relationship towards individual MHL dimensions.

Several limitations can be identified within this study. Due to the use of a convenience sampling method and the lack of random allocation to intervention and control group, it is not possible to reliably generalize the outcomes of this study. Moreover, due to the relatively small sample size, it was not possible to conduct sub-group or multivariate analysis to identify factors that might have further supported the observed changes in HSE and MHK. For example, it would have been worthwhile to investigate whether there are any differences in the outcomes of the intervention in regards to gender, or socio-economic background, or in the two different implementation modalities (in-person or video contact) of the module related to lived experience of mental illness. Another limitation related to the study design is the impossibility of assessing actual help-seeking behavior as well as general stability of the results over time due to the lack of a longitudinal follow-up assessment. The outcome measures used for MHK and HSE are not standardized instruments and have been translated for the purpose of this evaluation study by our research team. In the absence of a rigorous psychometric evaluation of the (German) MHK and HSE scale it is not clear how accurate the constructs of mental health knowledge and help-seeking efficacy are depicted. In this study, the MHK and HSE reached acceptable internal consistency scores at baseline and post-test. While the MHK scale has previosuly been found to contain various domains of knowledge, the HSE scale rather seems to load on a single factor, even though it combines help-seeking efficacy for oneself and with regards to others (39). In-depth psychometric testing is warranted to assess the performance of these instruments.

The strengths of the current research endeavor lie in using an already existing evidence-based resource and further extending its realm of application to German-speaking countries. This provides the possibility to further investigate and identify universally applicable elements across different countries and contexts. Given that schools and colleges have been identified as relevant mental health promotion settings for young people (6), this resource brings added value to the context of school-based interventions in Germany. Moreover, it complements as well as goes beyond the scope of other similar intervention efforts in Germany [such as “Crazy? So what” (45) or “Mind Matters” (46)], which have mainly been aimed at destigmatizing mental illness and promoting mental health, but have not explicitly targeted MHL. Furthermore, while most studies verified its effectiveness as part of weekly scheduled classes, this study confirms its usability as part of a one-day intervention. While a one-day intervention might be a more easily manageable option for some schools, it should be noted, that the long-term effectiveness of the MHC has so far only been confirmed for its weekly application as part of regular classes (33).

Finally, we argue that due to the multifactorial cause of the emergence of mental illness, a mental health promotion perspective would require interventions to take place on the continuum of the individual to structural level (6). The findings of this study are situated at the individual level. While this universal, youth-friendly approach has proven helpful in strengthening relevant dimensions of MHL, it is still necessary to specifically tackle risk factors of mental illness. In the “social determinants of (mental) health” framework, social inequality is considered to be the root cause for factors that negatively impact mental health (such as unemployment, occupational social class, precarious working conditions, or neighborhood safety) (9). Thus, a structural strategy would be to decrease social inequalities within and between nations. In order to achieve a proper and comprehensive public mental health promotion response, interventions at individual and structural levels are necessary.

In conclusion, MHK and HSE of 10^th^ grade students were significantly improved after participating in a MHL-based intervention that was adapted and translated for the German school setting. These are the first promising insights in the usefulness and applicability of an already established MHL resource for Germany, and pave the way for a broad roll out of the intervention and future effectiveness studies.

## Data availability statement

The raw data supporting the conclusions of this article will be made available by the authors, without undue reservation.

## Ethics statement

The studies involving human participants were reviewed and approved by ethikkommission@uni-bielefeld.de. Written informed consent to participate in this study was provided by the participants’ legal guardian/next of kin.

## Author contributions

AF and SK contributed to the conceptualization, methodology, investigation, data curation, and analysis. AF wrote the first draft of the manuscript. AF and SK contributed to the data visualization, editing, and reviewing of the manuscript. UB and OO contributed to the reviewing of the manuscript, funding acquisition, conception, and study design. All authors contributed to the article and approved the submitted version.

## Funding

This research was funded by the German Federal Ministry of Education and Research, grant numbers 01EL1824A and 01EL1824F. The authors acknowledge support for the publication costs by the Open Access Publication Fund of Bielefeld University and the Deutsche Forschungsgemeinschaft (DFG).

## Conflict of interest

The authors declare that the research was conducted in the absence of any commercial or financial relationships that could be construed as a potential conflict of interest.

## Publisher’s note

All claims expressed in this article are solely those of the authors and do not necessarily represent those of their affiliated organizations, or those of the publisher, the editors and the reviewers. Any product that may be evaluated in this article, or claim that may be made by its manufacturer, is not guaranteed or endorsed by the publisher.

## Footnotes

^1^The cited study verified this for depression.

^2^Material hardship refers to the lack of resources for meeting basic needs such as not having enough food or having utilities turned off because of inability to pay bills.

^3^The post-test was planned for 1 to 2 weeks after finalization of the MHC, but could be carried out only 12 to 24  weeks after.

^4^School staff that provides psychological counseling.
