# Supplementary material for: The effects of an adapted mental health literacy curriculum for secondary school students in Germany on mental health knowledge and help-seeking efficacy: results of a quasi-experimental pre-post evaluation study
Source: Front Public Health. 2023 Aug 16;11:1219925. doi: 10.3389/fpubh.2023.1219925 (PMC10468570; doi:10.3389/fpubh.2023.1219925)
Supplement: Supplementary file 1 [file Table_1.DOCX]

Supplementary Material

The effects of an adapted mental health literacy curriculum for secondary school students in Germany on mental health knowledge and help-seeking efficacy: results of a pre-post evaluation study

Alexandra Maria Freţian^*^, Sandra Kirchhoff, Ullrich Bauer, Orkan Okan

*** Correspondence:**Alexandra Maria Freţian
fretian@uni-bielefeld.de

Table 1 Items of the mental health knowledge scale

| **Original English items** | **German translation** |
| --- | --- |
| 1. Mental health and mental illness both involve the brain and how it functions. | Psychische Gesundheit und psychische Erkrankung haben beide mit dem Gehirn und wie es funktioniert zu tun. (R) |
| 2. People who have mental illness can at the same time have mental health. | Menschen, die eine psychische Erkrankung haben, können zur gleichen Zeit psychisch gesund sein. (R) |
| 3. The brain can affect the way the body functions but the body can not affect the way the brain functions | Das Gehirn kann beeinflussen, wie der Körper funktioniert, aber der Körper kann nicht beeinflussen, wie das Gehirn funktioniert. (F) |
| 4. Different brain functions are all controlled by various neuron circuits forming networks communicating with each other. | *Schwerer und wiederkehrender Stress kann sich auf das Gehirn auswirken*.^1^(R) |
| 5. Most people who experience traumatic events such as a car accident will develop a post traumatic stress disorder. | Die meisten Personen, die traumatische Ereignisse erleben, wie z.B. ein Autounfall, werden eine Posttraumatische Belastungsstörung entwickeln. (F) |
| 6. Every person’s mood can fluctuate up and down naturally. | Die Stimmung von jeder Person kann normalerweise hoch und runter schwanken. (R) |
| 7. The brain acts to help control the functioning of the heart, lungs, and fingers. | Das Gehirn hilft dabei, die Funktionen von Herz, Lunge und der Finger zu kontrollieren. (R) |
| 8. Both genetic problems and infections can cause the brain to get sick and stop functioning normally. | Beides, genetische Probleme und Infektionen, können das Gehirn krank machen und dazu beitragen, dass es nicht mehr richtig funktioniert. (R) |
| 9. Cognition, body movements and signaling are ALL functions controlled by the brain. | Denken, körperliche Bewegungen und Signalverarbeitung sind ALLES Funktionen, die durch das Gehirn kontrolliert werden. (R) |
| 10. Feelings are controlled mostly by the heart. | Gefühle werden größtenteils vom Herz gesteuert. (F) |
| 11. Most people who have a mental illness don’t get well and stay well with treatment. | Die meisten Menschen, die eine psychische Erkrankung haben, werden mit einer Behandlung weder gesund werden noch gesund bleiben. (F) |
| 12. Vitamins and meditation are good treatments for most mental illnesses. | Vitamine und Meditation sind gute Behandlungen für die meisten psychischen Erkrankungen. (F) |
| 13. People who have schizophrenia often get a split personality. | Menschen, die eine Schizophrenie haben, bekommen häufig eine gespaltene Persönlichkeit. (F) |
| 14. Depression and Bipolar Disorder are two examples of the type of mental illnesses called mood disorders. | Depression und Bipolare Störung sind zwei Beispiele für psychische Erkrankungen, die mit der Stimmung zu tun haben. (R) |
| 15. An anxiety disorder happens when a person’s brain detects the presence of danger – such as a dog attacking. | Eine Angststörung tritt auf, wenn das Gehirn einer Person eine Gefahr entdeckt – wie z.B. ein angreifender Hund. (F) |
| 16. Panic attacks in Panic Disorder happen as a result of stresses in the environment. | Panikattacken einer Panikstörung geschehen als Folge von Stress im Umfeld. (F) |
| 17. People with social anxiety disorder often feel as if they are being scrutinized and judged by others. | Menschen mit sozialer Angststörung fühlen sich oft so, als würden sie von anderen beobachtet und verurteilt werden. (R) |
| 18. An SSRI medicine and cognitive behavioral therapy are given together to effective treat Obsessive Compulsive Disorder. | *Medikamente sollten nie genutzt werden, um eine psychische Erkrankung zu behandeln.*^2^ (F) |
| 19. Attention Deficit Hyperactivity Disorder has three components including attention problems, hyperactivity, and anxiety. | Die Aufmerksamkeitdefizits-Hyperaktivitätsstörung (ADHS) beinhaltet drei Komponenten: Probleme der Aufmerksamkeit, Hyperaktivität und Angst. (F) |
| 20. Suicide in young people is mostly the result of the stress of being a teenager. | Selbstmord bei jungen Menschen ist meistens die Folge vom Stress ein Jugendlicher zu sein. (F) |
| 21. Self-harming behaviors are the same as suicide attempts. | Selbstverletzende Verhaltensweisen sind dasselbe wie Selbstmordversuche. (F) |
| 22. Treatment of mental disorders has three purposes including, relieving symptoms, restoring functioning, and promoting recovery. | Die Behandlung von psychischen Störungen hat drei Ziele: die Symptome zu verringern, die Aufgaben des Alltags wieder bewältigen zu können und die Genesung zu fördern. (R) |
| 23. Mental illnesses are caused by usual stresses of everyday life. | Psychische Erkrankungen werden durch Alltagsstress verursacht. (F) |
| 24. All mental distress will develop into mental illness overtime. | Jede psychische Belastung wird sich mit der Zeit zu einer psychischen Erkrankung entwickeln. (F) |
| 25. Mental health can be improved by leading a physically healthy life. | Psychische Gesundheit kann durch eine körperlich gesunde Lebensweise verbessert werden. (R) |
| 26. If a person feels sad for a few days in a row, they likely have a Depression. | Wenn sich eine Person ein paar Tage hintereinander traurig fühlt, hat sie wahrscheinlich eine Depression. (F) |
| 27. Young people with Bulimia Nervosa often starve themselves and exercise excessively. | Junge Menschen mit Bulimie hungern oft und trainieren übermäßig viel. (F) |
| 28. Good social relationships and exercise BOTH help to promote good mental health. | Gute soziale Beziehungen und Bewegung helfen BEIDE die psychische Gesundheit zu fördern. (R) |
| 29. Occasional sadness and anger are signs of poor mental health. | Gelegentliche Traurigkeit und Wut sind Anzeichen für eine schlechte psychische Gesundheit. (F) |
| 30. The phenomenon of craving drives substance abuse. | Das Phänomen der Sucht fördert den Substanzmissbrauch. (F) |
| ^1^Original item: “Severe and repeated stress can affect the brain” from KAHM Scale (Simkiss, N.J.; Gray, N.S.; Dunne, C.; Snowden, R.J. Development and psychometric properties of the Knowledge and Attitudes to Mental Health Scales (KAMHS): a psychometric measure of mental health literacy in children and adolescents. *BMC Pediatr.* 2021, *21*, 508, doi:10.1186/s12887-021-02964-x)  ^2^ Original item: “Medicines should never be used to treat a mental disorder” from a different version of the Student Evaluation Survey of the [Mental Health High School Curriculum Guide](http://mentalhealthliteracy.org/schoolmhl/wp-content/uploads/2015/09/Mental-Health-High-School-Curriculum-Guide.pdf)  F = false statements  R = correct statements | |

Table 2 Items of the help-seeking efficacy scale

| **Original English items** | **German translation** |
| --- | --- |
| 1. In general, asking for help for a mental health problem or disorder is helpful. | Im Allgemeinen ist es bei einem psychischen Problem oder einer psychischen Erkrankung hilfreich, nach Hilfe zu fragen. |
| 2. I am comfortable asking for help for a mental health problem or disorder. | Ich fühle mich wohl, bei einem psychischen Problem oder einer psychischen Erkrankung nach Hilfe zu fragen. |
| 3. If I think I may have a mental health problem or mental disorder (such as depression, social anxiety disorder, etc), I will ask for help. | Wenn ich denke, dass ich ein psychisches Problem oder eine psychische Erkrankung haben könnte (wie Depression, Soziale Angststörung etc.), frage ich nach Hilfe. |
| 4. If I thought one of my friends or peers needed help with a mental health problem or disorder (such as depression), I would encourage them to seek help. | Wenn ich denken würde, dass einer meiner Freundinnen/Freunde oder Gleichaltrigen wegen einem psychischen Problem oder einer psychischen Erkrankung (wie Depression) Hilfe braucht, würde ich sie/ihn ermutigen, Hilfe aufzusuchen. |
| 5. If I thought one of my family members needed help with a mental health problem or disorder (such as depression), would encourage them to seek help. | Wenn ich denken würde, dass einer meiner Familienmitglieder wegen einem psychischen Problem oder einer psychischen Erkrankung (wie Depression) Hilfe braucht, würde ich sie/ihn ermutigen, Hilfe aufzusuchen. |

Table 3 Results of paired sample t-tests for individual items of the mental health knowledge scale in the intervention group

| **Mental health knowledge items** |  | **% of correct answers at T1** | **% of correct answers at T2** | **% of change** | **Paired sample t-test** |
| --- | --- | --- | --- | --- | --- |
| 1. Mental health and mental illness both involve the brain and how it functions. (R) |  | 82.1 | 88.7 | 6.6 | t(105)=-1.468, p=.145 |
| 2. People who have mental illness can at the same time have mental health. (R) |  | 27.6 | 65.7 | 38.1 | t(104)=-6.235, **p=<.001** |
| 3. The brain can affect the way the body functions but the body can not affect the way the brain functions. (F) |  | 23.6 | 34.9 | 11.3 | t(105)=-2.029, **p=.045** |
| 4. Severe and repeated stress can affect the brain.^1^ (R) |  | 93.4 | 95.2 | 1.8 | t(104)=-.576, p=.566 |
| 5. Most people who experience traumatic events such as a car accident will develop a post traumatic stress disorder. (F) |  | 17.9 | 30.2 | 12.3 | t(105)=-2.386, **p=.019** |
| 6. Every person’s mood can fluctuate up and down naturally. (R) |  | 89.6 | 84.9 | 4.7 | t(105)=-1.215, p=.277 |
| 7. The brain acts to help control the functioning of the heart, lungs, and fingers. (R) |  | 83.0 | 85.8 | 2.8 | t(105)=-.624, p=.534 |
| 8. Both genetic problems and infections can cause the brain to get sick and stop functioning normally. |  | 61.3 | 66.0 | 4.7 | t(105)=-.821, p=.414 |
| 9. Cognition, body movements and signaling are ALL functions controlled by the brain. (R) |  | 84.0 | 85.8 | 1.8 | t(105)=-.446, p=.657 |
| 10. Feelings are controlled mostly by the heart. (F) |  | 70.8 | 77.4 | 6.6 | t(105)=-1.713, p=.090 |
| 11. Most people who have a mental illness don’t get well and stay well with treatment. (F) |  | 53.3 | 73.6 | 20.3 | t(104)=-3.543, **p=<.001** |
| 12. Vitamins and meditation are good treatments for most mental illnesses. (F) |  | 27.6 | 31.4 | 3.8 | t(103)=-.754, p=.452 |
| 13. People who have schizophrenia often get a split personality. (F) |  | 12.3 | 32.4 | 20.1 | t(104)=-4.037, **p=<.001** |
| 14. Depression and Bipolar Disorder are two examples of the type of mental illnesses called mood disorders. (R) |  | 76.4 | 93.3 | 16.9 | t(104)= -3.760, **p=<.001** |
| 15. An anxiety disorder happens when a person’s brain detects the presence of danger – such as a dog attacking. (F) |  | 34.0 | 45.3 | 11.3 | t(105)=-2.029, **p=.045** |
| 16. Panic attacks in Panic Disorder happen as a result of stresses in the environment. (F) |  | 10.4 | 27.4 | 17 | t(105)=-3.587, **p=<.001** |
| 17. People with social anxiety disorder often feel as if they are being scrutinized and judged by others. (R) |  | 80.2 | 93.4 | 13.2 | t(105)=-3.467, **p=<.001** |
| 18. Medicines should never be used to treat a mental disorder. (F) |  | 59.4 | 90.6 | 31.2 | t(105)=-5.925, **p=<.001** |
| 19. Attention Deficit Hyperactivity Disorder has three components including attention problems, hyperactivity, and anxiety. (F) |  | 35.2 | 45.3 | 10.1 | t(104)= -1.882, p=.063 |
| 20. Suicide in young people is mostly the result of the stress of being a teenager. (F) |  | 48.1 | 54.7 | 6.6 | t(105)= -1.304, p=.195 |
| 21. Self-harming behaviors are the same as suicide attempts. (F) |  | 70.5 | 70.8 | 0.3 | t(104)= -.208, p=.836 |
| 22. Treatment of mental disorders has three purposes including, relieving symptoms, restoring functioning, and promoting recovery. (R) |  | 93.4 | 91.7 | 1.7 | t(105)=.631, p=.530 |
| 23. Mental illnesses are caused by usual stresses of everyday life. (F) |  | 35.6 | 51.9 | 16.3 | t(103)= -2.811, **p=.006** |
| 24. All mental distress will develop into mental illness overtime. (F) |  | 50.9 | 79.2 | 28.3 | t(105)= -5.496, **p=<.001** |
| 25. Mental health can be improved by leading a physically healthy life. (R) |  | 59.4 | 84.9 | 25.5 | t(105)=-4.600, **p=<.001** |
| 26. If a person feels sad for a few days in a row, they likely have a Depression. (F) |  | 87.6 | 89.6 | 2 | t(104)= -.631, p=.530 |
| 27. Young people with Bulimia Nervosa often starve themselves and exercise excessively. (F) |  | 23.6 | 40.6 | 17 | t(105)= -3.121, **p=.002** |
| 28. Good social relationships and exercise BOTH help to promote good mental health. (R) |  | 82.1 | 92.5 | 10.4 | t(105)= -2.591, **p=.011** |
| 29. Occasional sadness and anger are signs of poor mental health. (F) |  | 69.8 | 82.1 | 12.3 | t(105)= -2.386, **p=.019** |
| 30. The phenomenon of craving drives substance abuse. (F) |  | 58.5 | 68.9 | 10.4 | t(105)= -2.241, **p=.027** |
| ^1^ from KAHM Scale (Simkiss, N.J.; Gray, N.S.; Dunne, C.; Snowden, R.J. Development and psychometric properties of the Knowledge and Attitudes to Mental Health Scales (KAMHS): a psychometric measure of mental health literacy in children and adolescents. BMC Pediatr. 2021, 21, 508, doi:10.1186/s12887-021-02964-x)  ^2^ Original item: “Medicines should never be used to treat a mental disorder” from a different version of the Student Evaluation Survey of the [Mental Health High School Curriculum Guide](http://mentalhealthliteracy.org/schoolmhl/wp-content/uploads/2015/09/Mental-Health-High-School-Curriculum-Guide.pdf)  F = false statements  R = correct statements | | | | | |
